# Supplementary material for: Isoform specific anti-TGFβ therapy enhances antitumor efficacy in mouse models of cancer
Source: Commun Biol. 2021 Nov 17;4:1296. doi: 10.1038/s42003-021-02773-z (PMC8599839; doi:10.1038/s42003-021-02773-z)
Supplement: Supplementary file 2 — Supplementary Information [file 42003_2021_2773_MOESM2_ESM.pdf]

Supplementary Figures for Isoform specific anti-TGFβ therapy enhances antitumor efficacy in mouse models of cancer

Aditi Gupta[1,2], Sadna Budhu[1,2], Kelly Fitzgerald[1,2], Rachel Giese[1,2], Adam O. Michel[3], Aliya Holland[1,2], Luis Felipe Campesato[1,2], Jacques van Snick[4], Catherine Uyttenhove[4], Gerd Ritter[5], Jedd D. Wolchok[1,2,6,7,8*] and Taha Merghoub[1,2,6,7,8*]

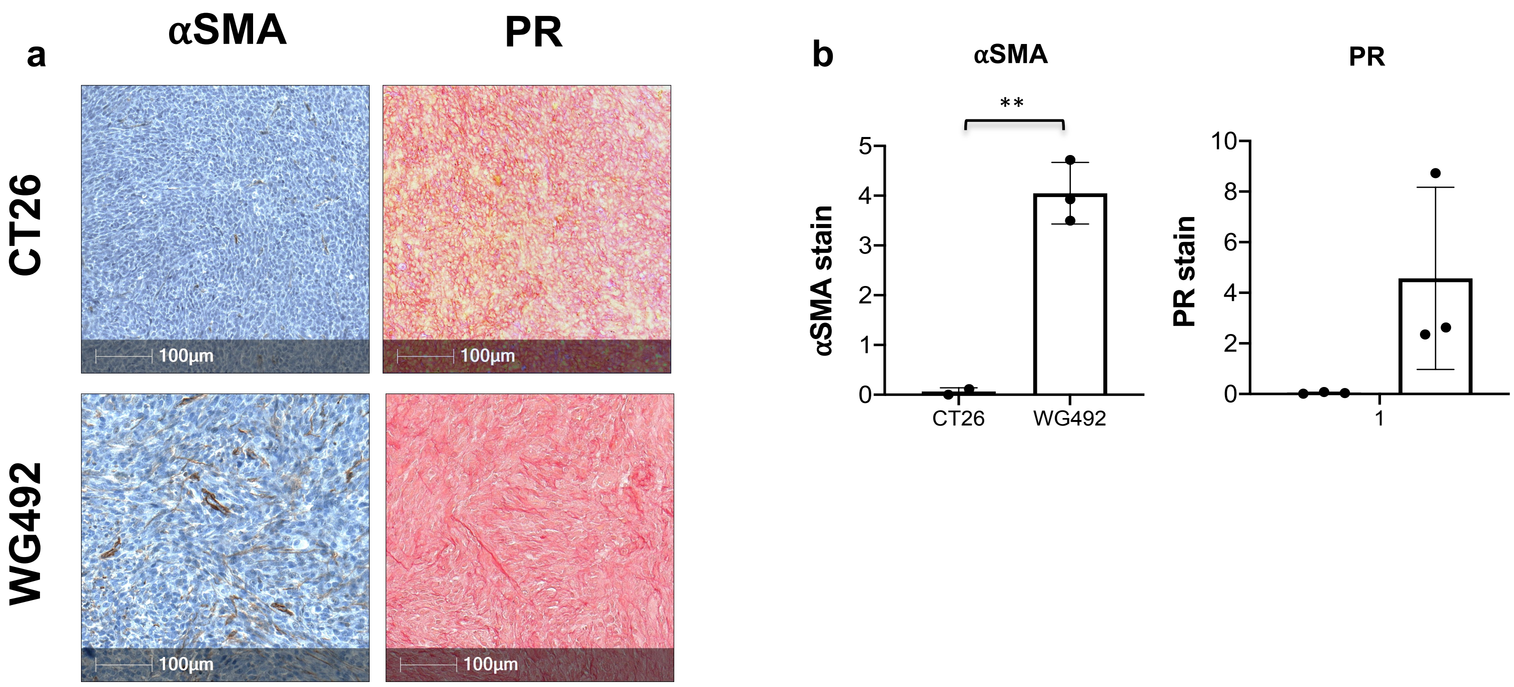

Supplementary figure 1: CT26 colon and WG492 melanoma as examples of stroma poor and stroma heavy murine tumor models, respectively

Syngeneic mice were implanted with 200,000 CT26 and 200,000 WG492 cells injected subcutaneously (n = 3 mice/ group). Tumors were harvested 10 days post tumor challenge and fixed for immunohistochemistry (IHC) prior to staining with picrosirus red (PR) and alpha-smooth muscle actin (αSMA). a) Representative cross sections of CT26 colon (top) and WG492 melanoma (bottom) stained for αSMA and PR. b) Bar graphs demonstrate quantification of the staining of either PR or αSMA $\pm$ standard error of the mean (SEM) following analysis by Halo software with supervision from a pathologist. **$p<0.005$

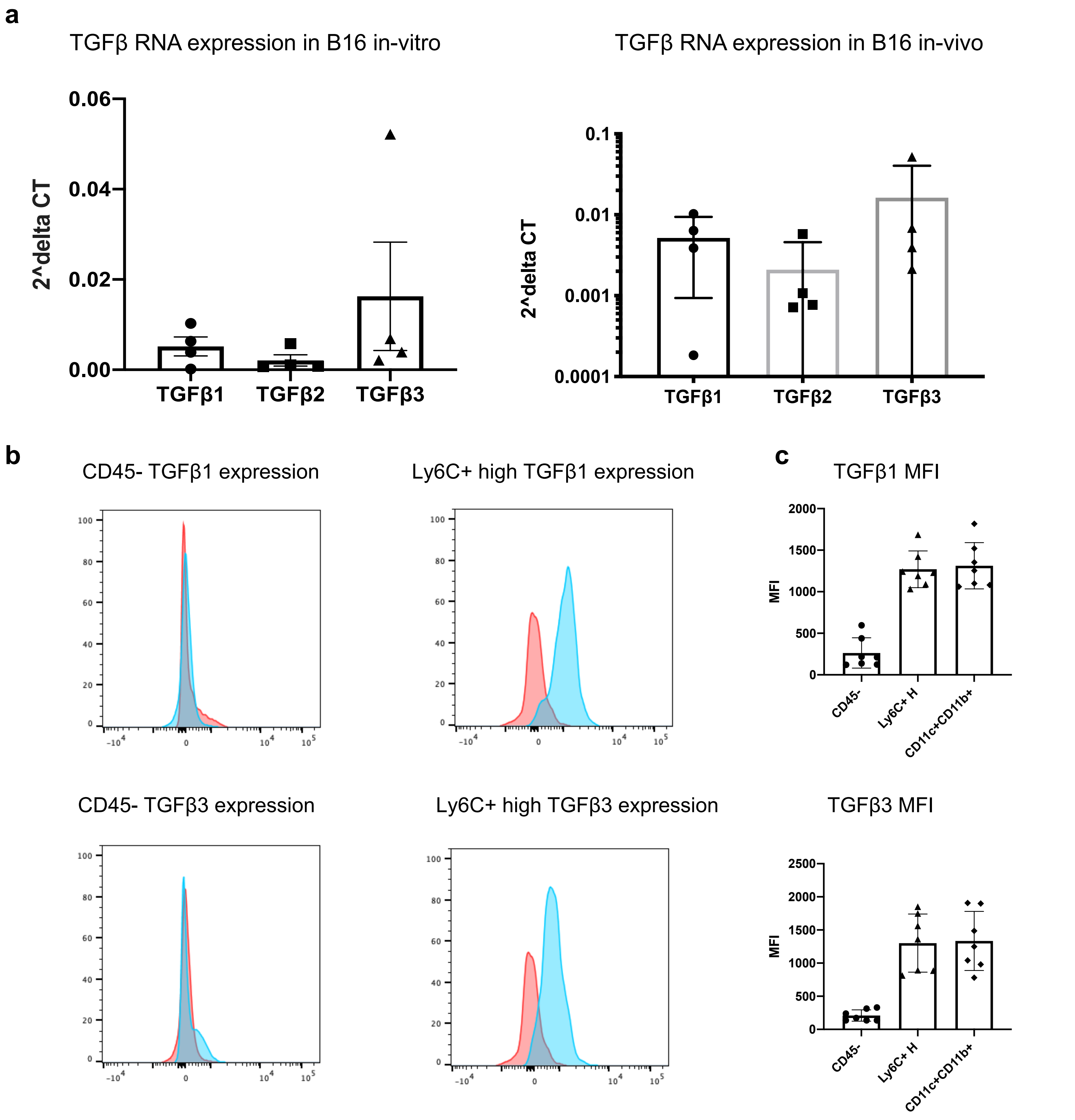

Supplementary figure 2: Immune cells are the main source of TGFβ in B16 tumors
a) mRNA expression of TGFβ isoforms detected via quantitative PCR in B16 tumor cells in-vitro (left) and in-vivo (right). For in-vivo analysis, tumors were harvested 11 days post implant and RNA was extracted. Data is shown as 2^delta CT relative to RNA expression of house keeping genes (GAPDH) and is plotted as mean ± standard deviation (SD) with n = 5 mice/ group. b) Representative plots showing relative expression of TGFβ1 and TGFβ3 on CD45- B16 tumor cells and Ly6C+ high monocytes based on MFI. The red peak represents the fluoresce minus one (FMO) or negative staining for each particular cell type and the blue peak represents positively staining cells. c) Relative MFI expression of TGFβ isoforms on immune cells compared to CD45- B16 tumor cells. Data shown is mean MFI ± standard deviation (SD) on indicated cell types with n = 7 mice/ group. Data is representative of two independent experiments.

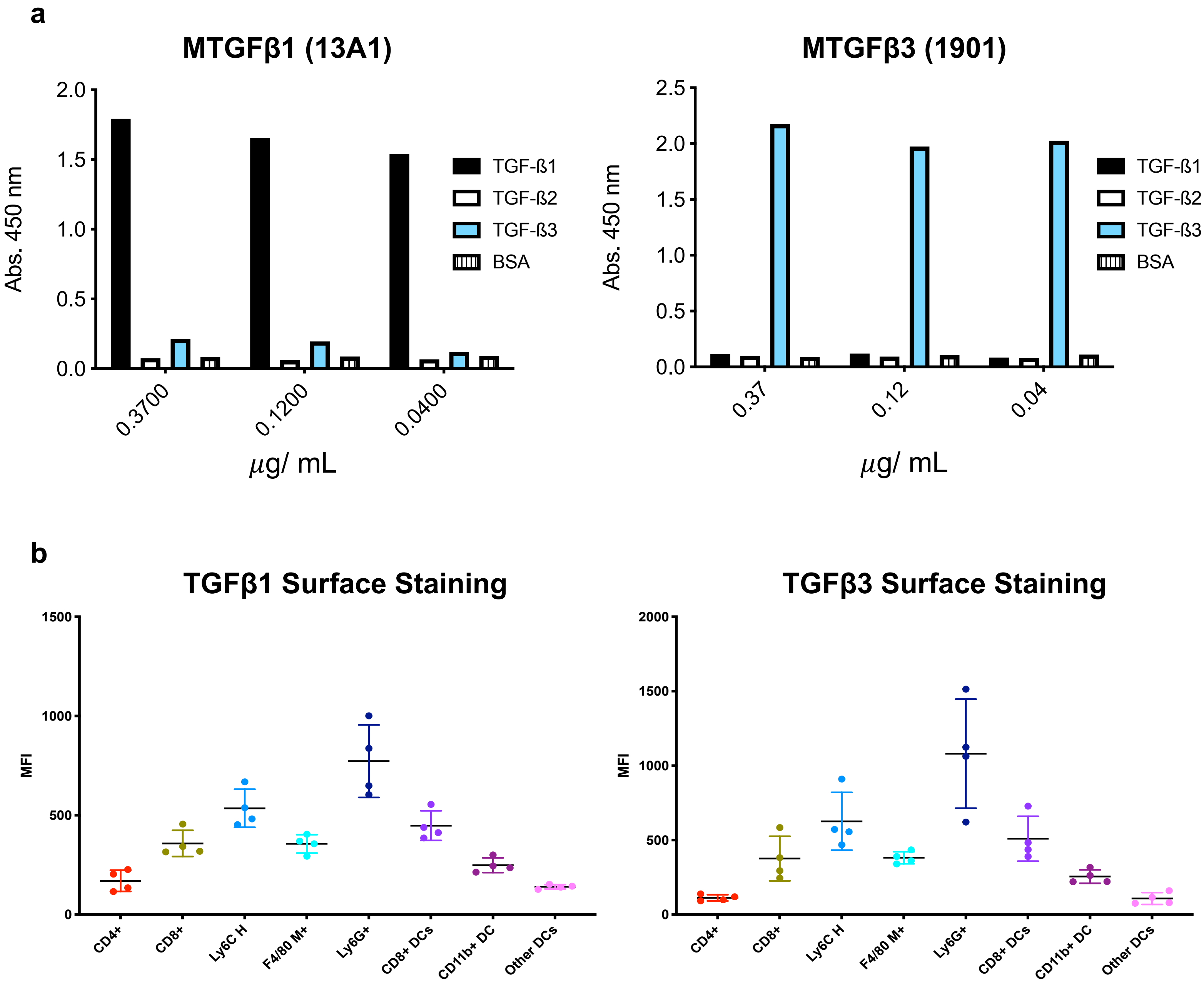

Supplementary figure 3: Binding specificities of TGFβ1 and TGFβ3 antibodies and cell surface staining of TGFβ isoforms on tumor infiltrating immune cells. a) The specificities of the anti-TGFβ1 monoclonal antibody clone 13A1 (IgG1) and anti-TGFβ3 monoclonal antibody clone 1901 (IgG1) were determined by ELISA. Shown are the absorbance at 450nm at the indicated concentrations of each protein tested. b) Surface staining of TGFβ1 and TGFβ3 isoforms on tumor infiltrating immune cells. B16 tumors were harvested from mice 11 days following tumor implantation. After creating single cell suspensions, tumors underwent processing and staining for flow cytometry analysis without fixation and permeabilization. Data represents mean fluorescence intensity (MFI) ± standard deviation (SD) of either TGFβ1 (left panel) or TGFβ3 (right panel) surface staining on specific tumor infiltrating immune cells (n = 5 mice/ group). Data is representative of two independent experiments.

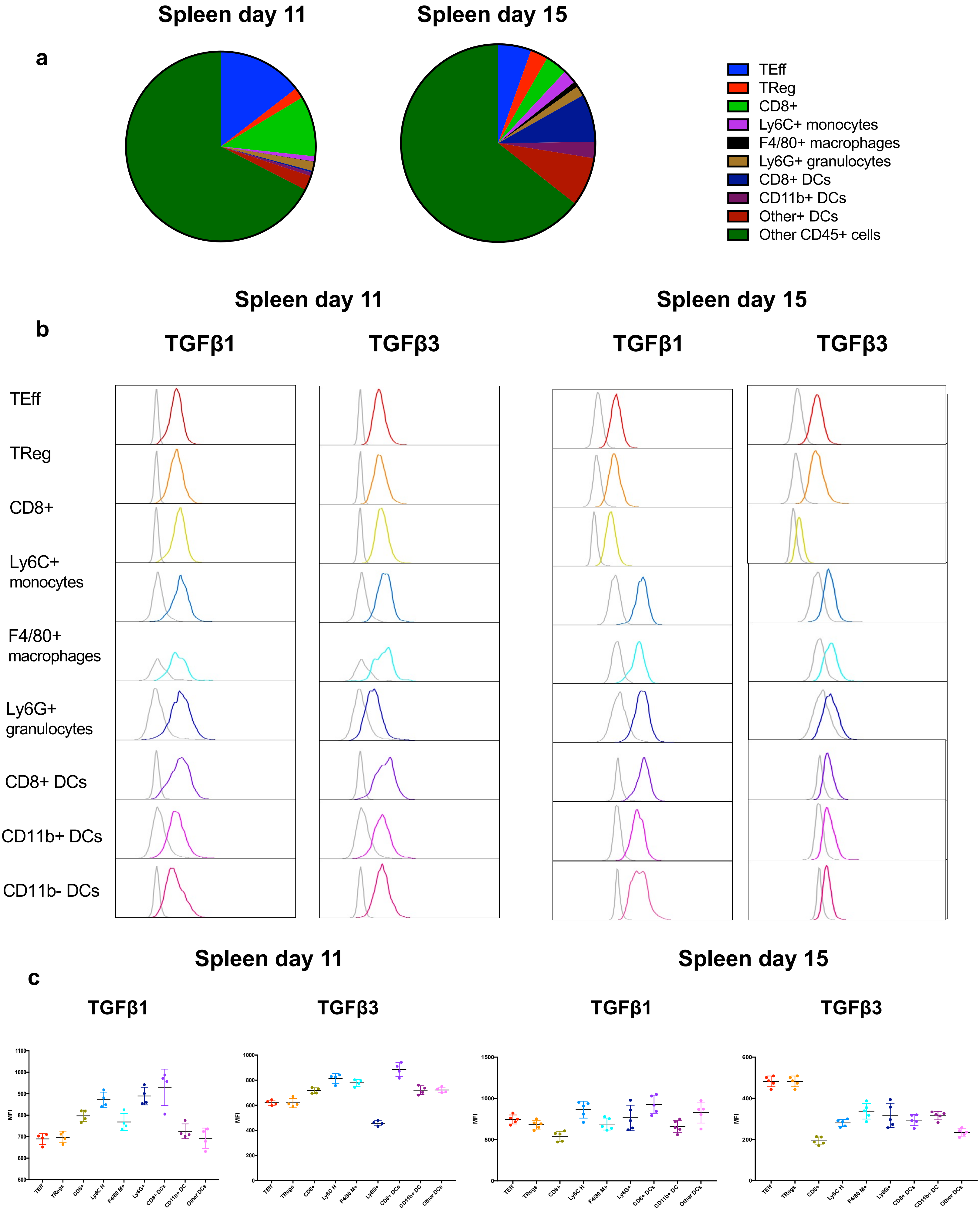

a
Spleen day 11
Spleen day 15
TEff
TReg
CD8+
Ly6C+ monocytes
F4/80+ macrophages
Ly6G+ granulocytes
CD8+ DCs
CD11b+ DCs
Other+ DCs
Other CD45+ cells
b
Spleen day 11
Spleen day 15
TGFβ1
TGFβ3
TGFβ1
TGFβ3
TEff
TReg
CD8+
Ly6C+ monocytes
F4/80+ macrophages
Ly6G+ granulocytes
CD8+ DCs
CD11b+ DCs
CD11b- DCs
c
Spleen day 11
Spleen day 15
TGFβ1
TGFβ3
TGFβ1
TGFβ3
MFI
TEff
TRegs
CD8+
Ly6C H
F4/80 M+
Ly6G+
CD8+ DCs
CD11b+ DC
Other DCs

Supplementary figure 4: Myeloid cells and dendritic cells (DCs) in the spleens of tumor challenged mice display the highest levels of TGFβ isoform expression

Spleens from mice bearing B16 tumors were harvested at 11 and 15 days following tumor implantation. After creating single cell suspensions, tumors underwent processing and staining for flow cytometry analysis as described in Materials and Methods. a) Breakdown of the immune infiltrate in B16 spleens of mice harvested 11 and 15 days after tumor implantation. b) Representative histograms displaying MFI of either TGFβ1 (left panel) or TGFβ3 (right panel) on immune cells in the spleens of tumor challenged mice as detected by flow cytometry. The light gray peak represents each cell type's FMO and was used to determine positive expression, indicated by the colored peak. c) Representative graph illustrating the relative expression of TGFβ1 or TGFβ3 by various lymphocytic and myeloid cell types in the spleen microenvironment 11 and 15 days after tumor implantation. Data (n = 5 mice/ group) is displayed as mean MFI $\pm$ SD. Data is representative of three independent experiments.

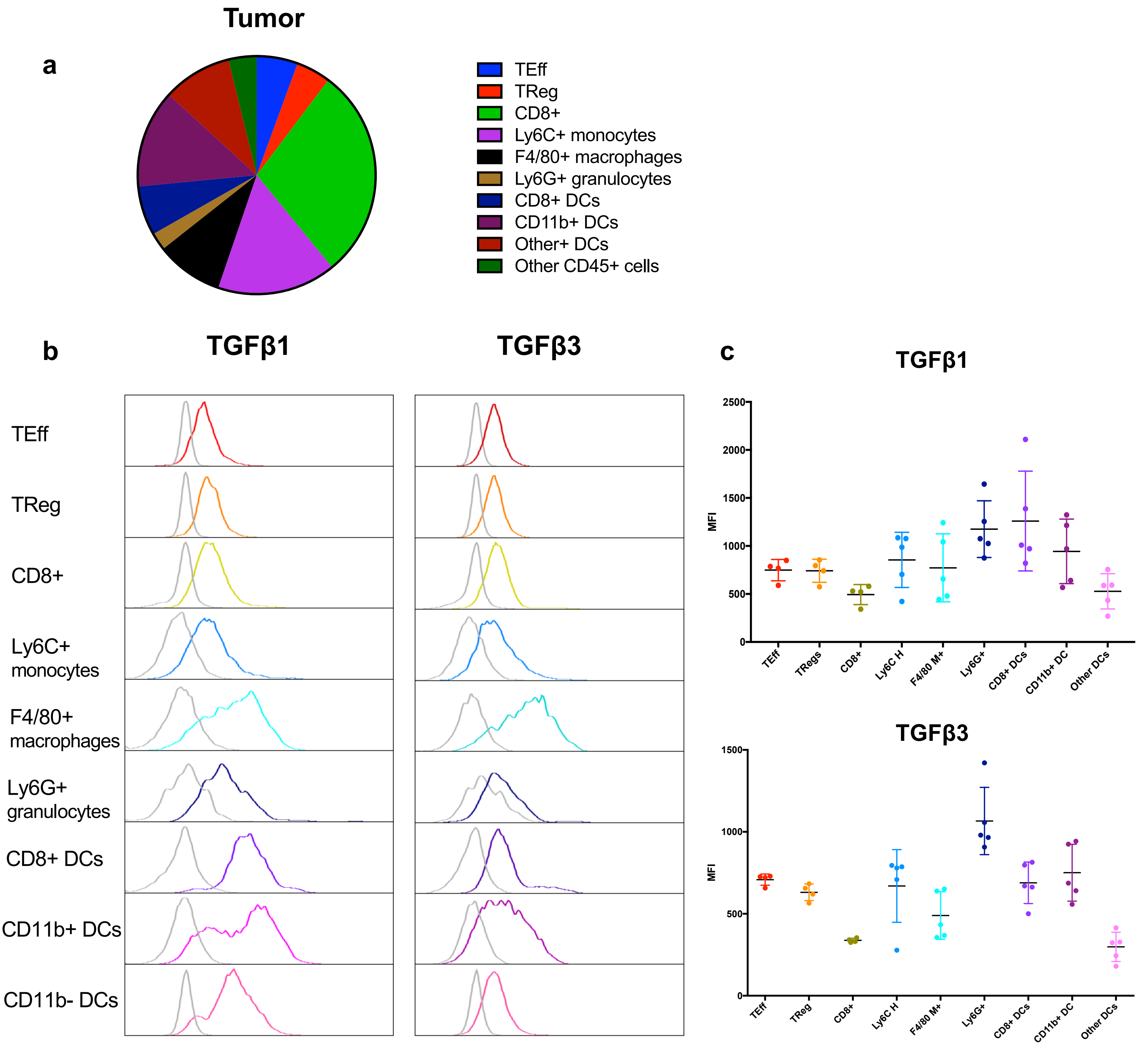

Supplementary figure 5: Tumor infiltrating myeloid immune cells and dendritic cells (DCs) display the highest levels of TGFβ isoform expression later on in tumor progression

B16 tumors from mice were harvested 15 days following tumor implantation. After creating single cell suspensions, tumors underwent processing and staining for flow cytometry analysis as described in Materials and Methods.

a) Breakdown of the immune infiltrate in B16 tumors of mice harvested 15 days after tumor implantation. b) Representative histograms displaying MFI of either TGFβ1 (left panel) or TGFβ3 (right panel) on specific tumor infiltrating immune cells as detected by flow cytometry. The light gray peak represents each cell type's FMO and was used to determine positive expression, indicated by the colored peak. c) Representative graph illustrating the relative expression of TGFβ1 (top graph) or TGFβ3 (bottom graph) by various lymphocytic and myeloid cell types in the tumor microenvironment 15 days after tumor implantation. Data (n = 5 mice/ group) is displayed as mean MFI ± SD. Data is representative of three independent experiments.

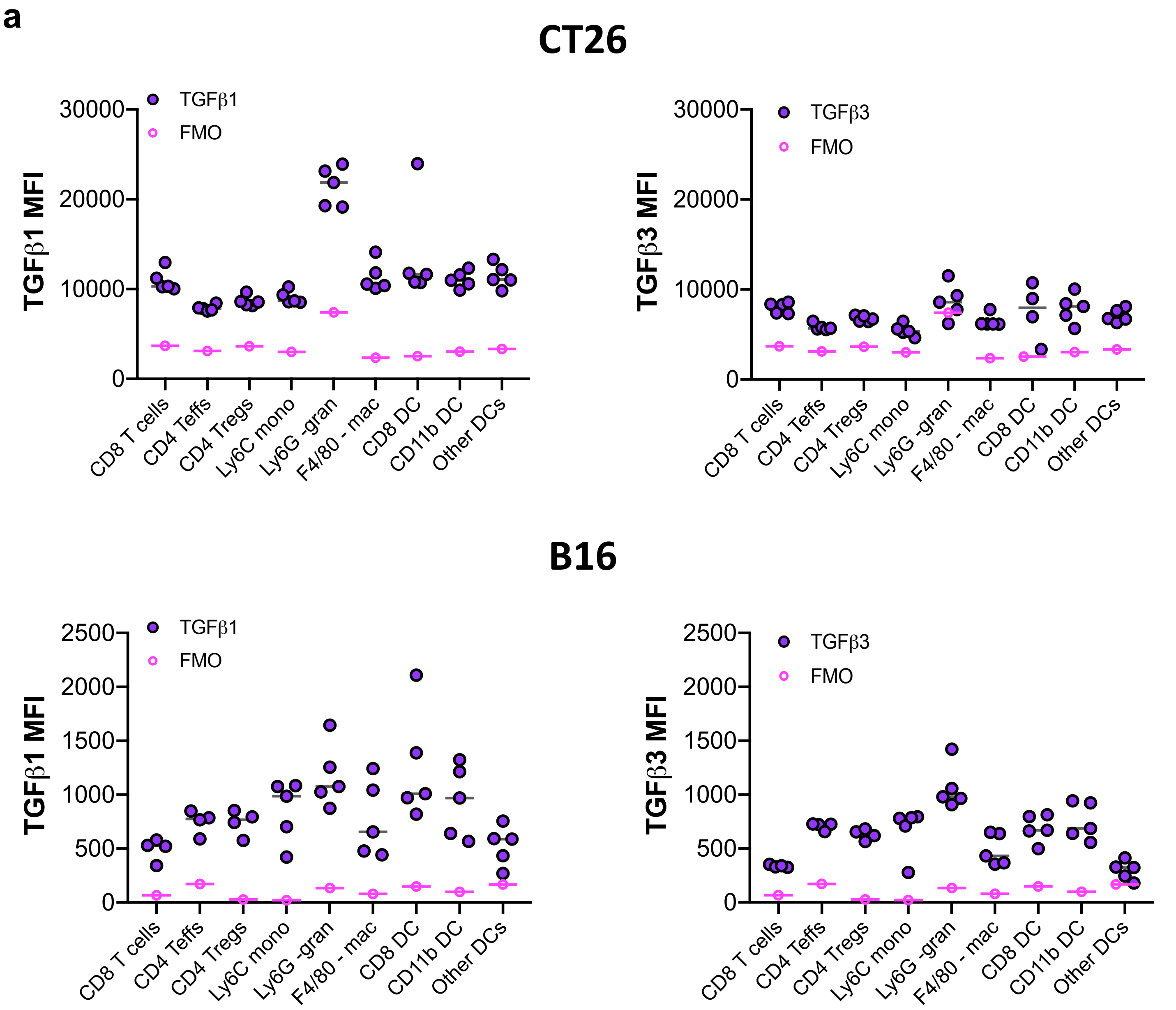

Supplementary figure 6: TGFβ isoform expression on tumor infiltrating immune cells in CT26 versus B16
200,000 CT26 tumor cells were implanted subcutaneously and 250,000 B16 cells were implanted intradermally into the right hind flank of syngeneic mice. Tumors were harvested 11 days following tumor implantation and processed as discussed in Materials and Methods for flow cytometry analysis. a) Plots showing relative MFI expression of TGFβ1 and TGFβ3 on infiltrating immune cells in CT26 (top) and B16 (bottom) tumors compared to the negative stain or fluorescence minus one (FMO). Data (n = 5 mice/ group) is displayed as mean MFI ± standard deviation (SD). Data is representative of two independent experiments.

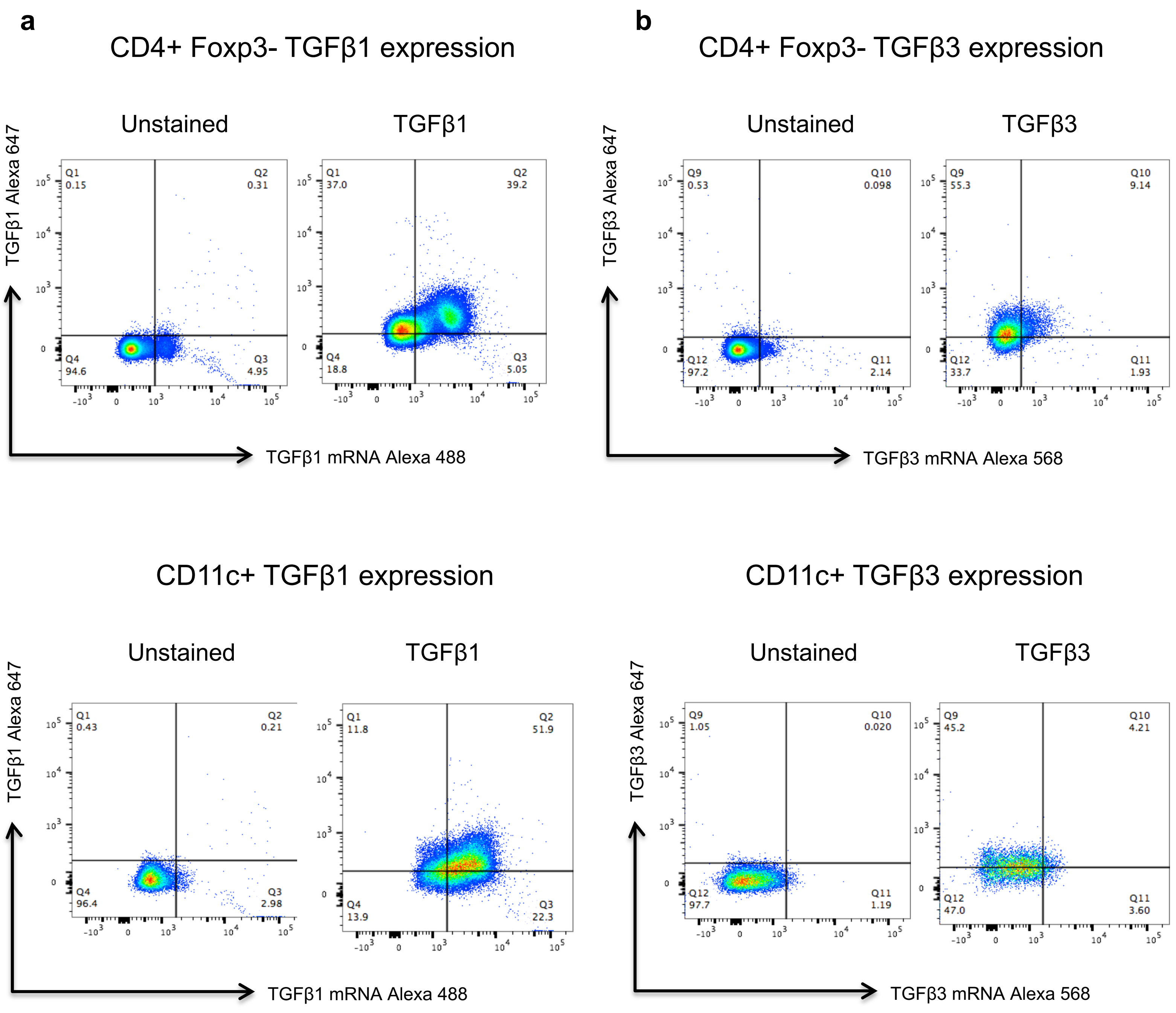

Supplementary figure 7: TGFβ mRNA detected by flow cytometry correlates with TGFβ protein expression on immune cells

Representative plots showing co-staining of TGFβ mRNA (x-axis) and TGFβ protein (y-axis) on CD4+Foxp3-T cells (TEff) and CD11c+ dendritic cells (DCs) from B16 tumors using PrimeFlow RNA assay (n = 10 mice/ group). Tumors were harvested 11 days following tumor challenge and underwent PrimeFlow protocol, followed by flow cytometric processing and analysis as described in Materials and Methods. Double positive cells were gated based off unstained cells. Data is representative of three independent experiments with 7 mice per group. a) Co-staining of TGFβ1 mRNA and TGFβ1 protein on TEffs and DCs. b) Co-staining of TGFβ3 mRNA and TGFβ3 protein on TEffs and DCs.

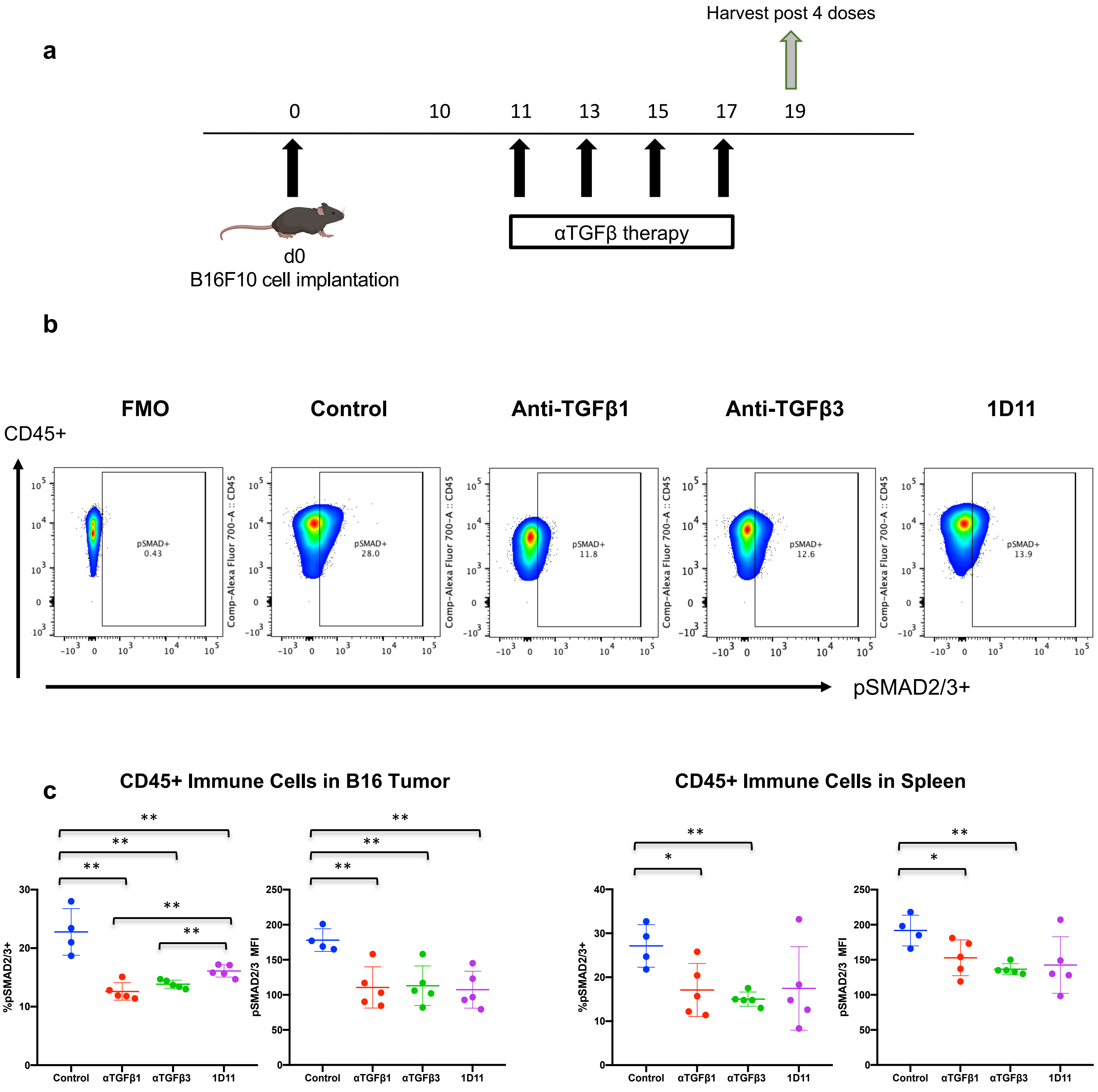

Supplementary figure 8: Inhibition of canonical TGFβ signaling following isoform specific and pan-TGFβ inhibition

a) Mice were implanted with 250,000 B16F10 cells and harvested according to the experimental schema shown. Tumors and spleens were processed and stained for flow cytometric analysis as detailed in Materials and Methods. b) Representative flow cytometry plots of phosphorylated SMAD2/3 (pSMAD2/3+) staining on CD45+ immune cells isolated from tumors of animals treated with isoform specific or pan-TGFβ inhibition. Gating of positive cells was based off of FMO (fluorescence minus one) or negative staining. c) Inhibition of canonical TGFβ signaling via reduced pSMAD2/3 detection on CD45+ immune cells isolated from tumors (left) and spleens (right) of animals treated with isoform specific or pan-TGFβ inhibition as demonstrated by MFI. Data shown represents mean MFI ± SD with n = 5 mice/ group. No difference in pSMAD2/3+ was detected on CD45- B16 tumor cells. pSMAD2/3+ cells were gated based off an FMO sample shown in panel B. Data shown is representative of two independent experiments. *$p<0.05$; **$p<0.005$

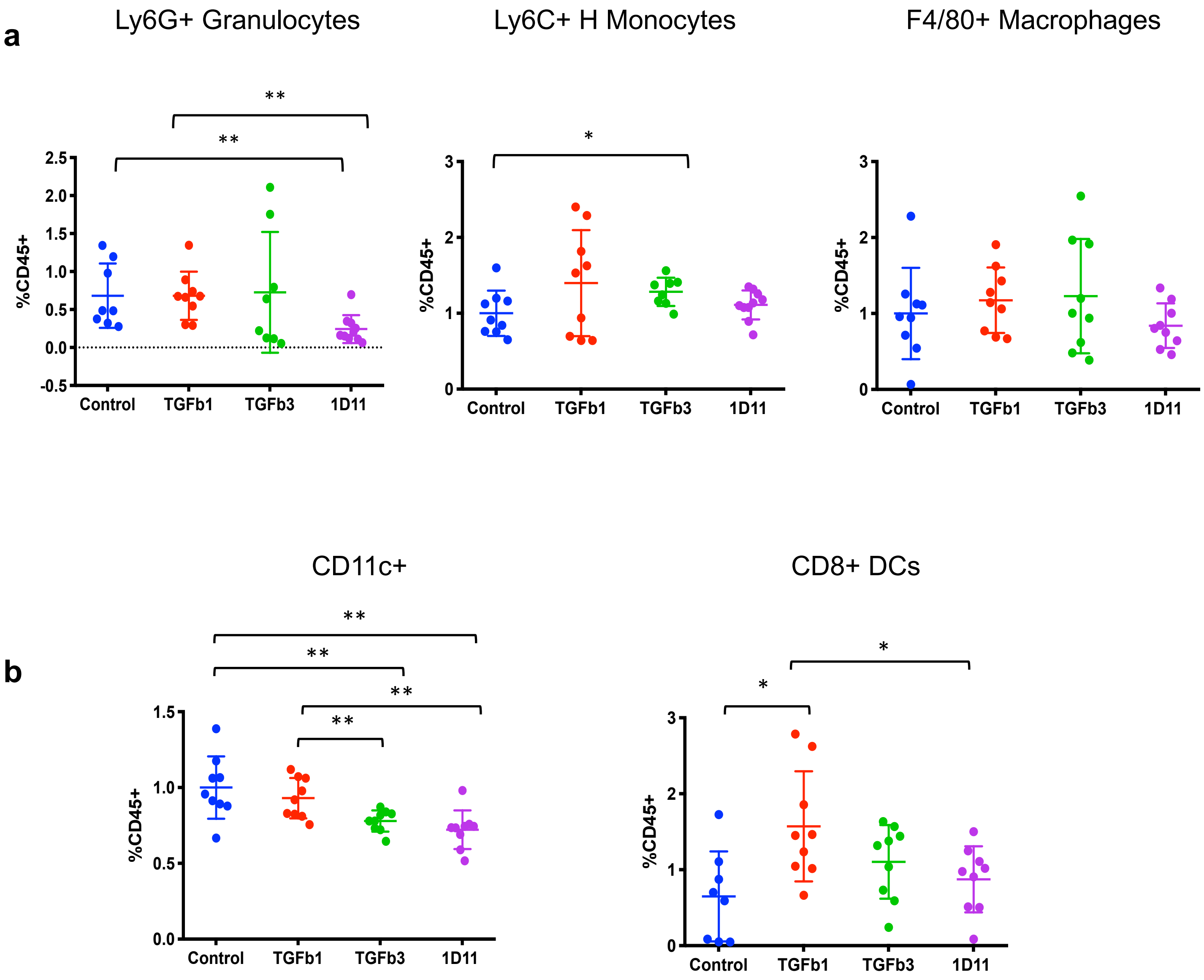

Supplementary figure 9: TGFβ inhibition does not effect myeloid infiltration of B16 tumors
B16 tumors from mice were harvested as shown in figure 4a following anti-TGFβ treatment and underwent processing for flow cytometric analysis as described in Material and Methods. A) Plots illustrating relative percentages of indicated myeloid cell types. Cells were gated based off CD45+CD11b+ and are plotted as a percentage of total CD45+ population within B16 tumors. B) Plots illustrating relative percentages of dendritic cells within B16 tumors. Cells were gated based off CD45+CD11c+ (left) and CD45+CD11c+MHCII+CD8+ (right) and are plotted as a percentage of total CD45+ population within B16 tumors. Data represents pooled values from two independent experiments normalized to the control (n = 5 mice/ group) and is displayed as fold change compared to the control ± SD gated on indicated cells types. *p<0.05; **p<0.005

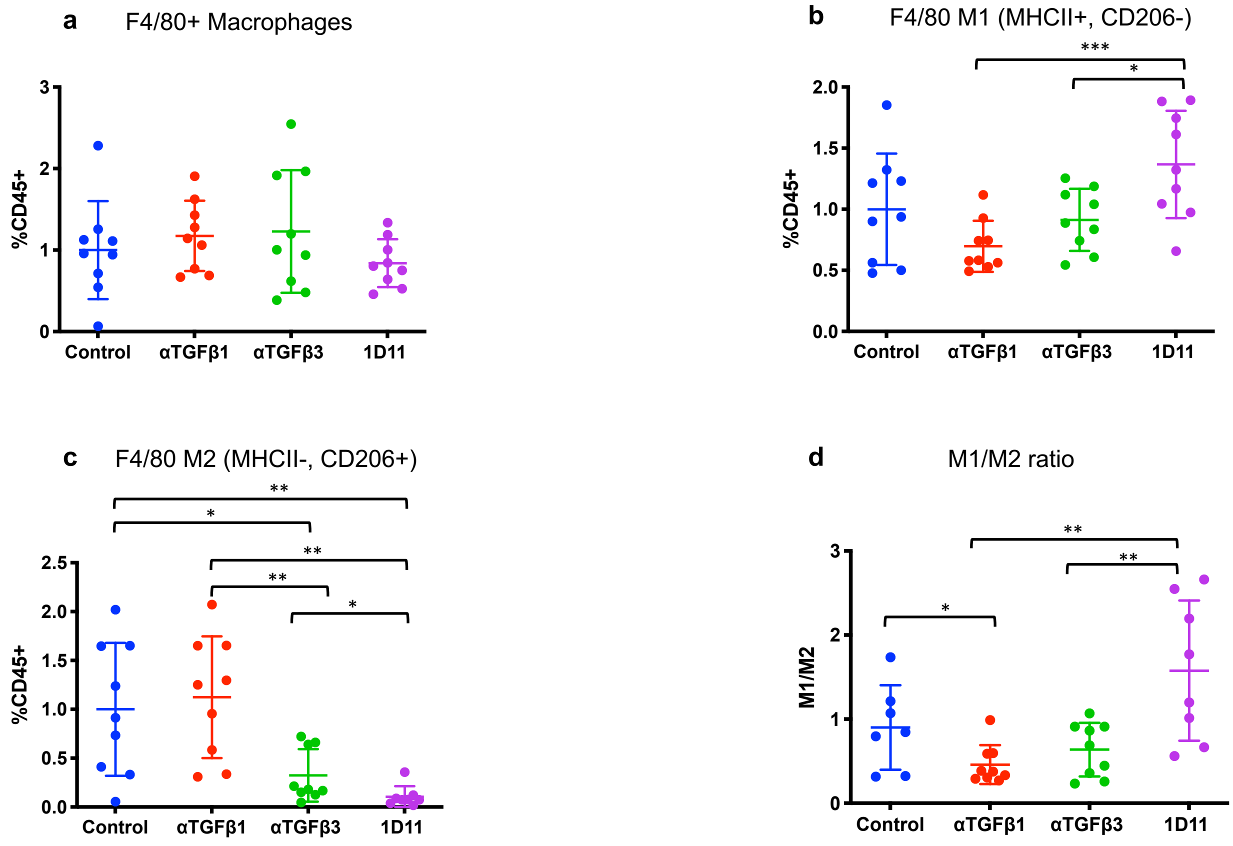

Supplementary figure 10: Isoform specific TGFβ inhibition has no effects on tumor infiltrating macrophage populations

B16 tumors were implanted into mice and harvested according to the scheme shown in Figure 4a. Tumors underwent processing for flow cytometry and analysis as discussed in Materials and Methods section. a) Plot showing F4/80+ macrophages harvested from animals treated with isoform specific versus pan-TGFβ inhibition. Gating was first done on CD45+ immune cells, followed by CD11b+ (excluding CD3+ T cells) and then F4/80+ macrophages. b) Plot showing M1 macrophage subpopulation defined as MHCII+, CD206-. c) Plot showing M2 macrophage subpopulation defined as MHCII-, CD206+. d) Plot shows the ratio of M1/M2 macrophages in each group of treated mice. Data represents pooled values from two independent experiments normalized to the control (n = 5 mice/ group) and is displayed as fold change compared to the control ± SD gated on indicated cells types. *p<0.05; **p<0.001; ***p<0.0005

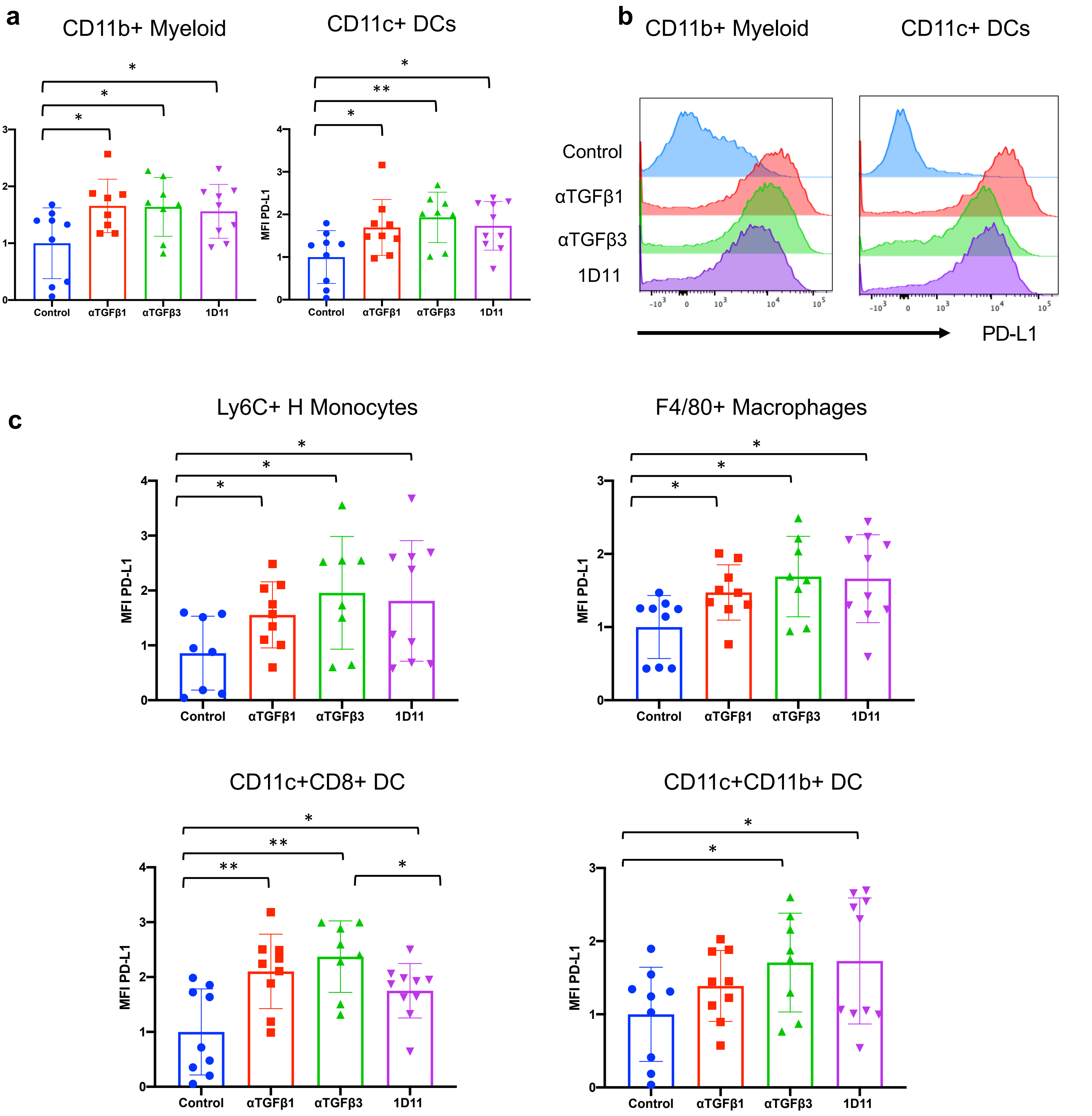

Supplementary figure 11: Enhanced PD-L1 expression on myeloid cells and dendritic cells following TGFβ inhibition

B16 tumors from mice were harvested as shown in figure 4a following anti-TGFβ treatment at day 19 post tumor implantation. a) Bar graphs showing PD-L1 expression on CD11b+ (left) and CD11c+ (right) tumor infiltrating myeloid cells 19 days post tumor implantation. b) Representative histograms showing the expression of PD-L1 on indicated cell types. c) Bar graphs showing PD-L1 expression on specific myeloid subtypes, Ly6C+ high monocytes and F4/80+ macrophages (top), and specific dendritic cell subtypes, CD8+ DCs and CD11b+ DCs (bottom). All data shown represents pooled MFI values from two independent experiments normalized to the control MFI (n = 5 mice/ group) and is displayed as fold change compared to the control MFI ± SD gated on indicated cells types. *p<0.05; **p<0.005.

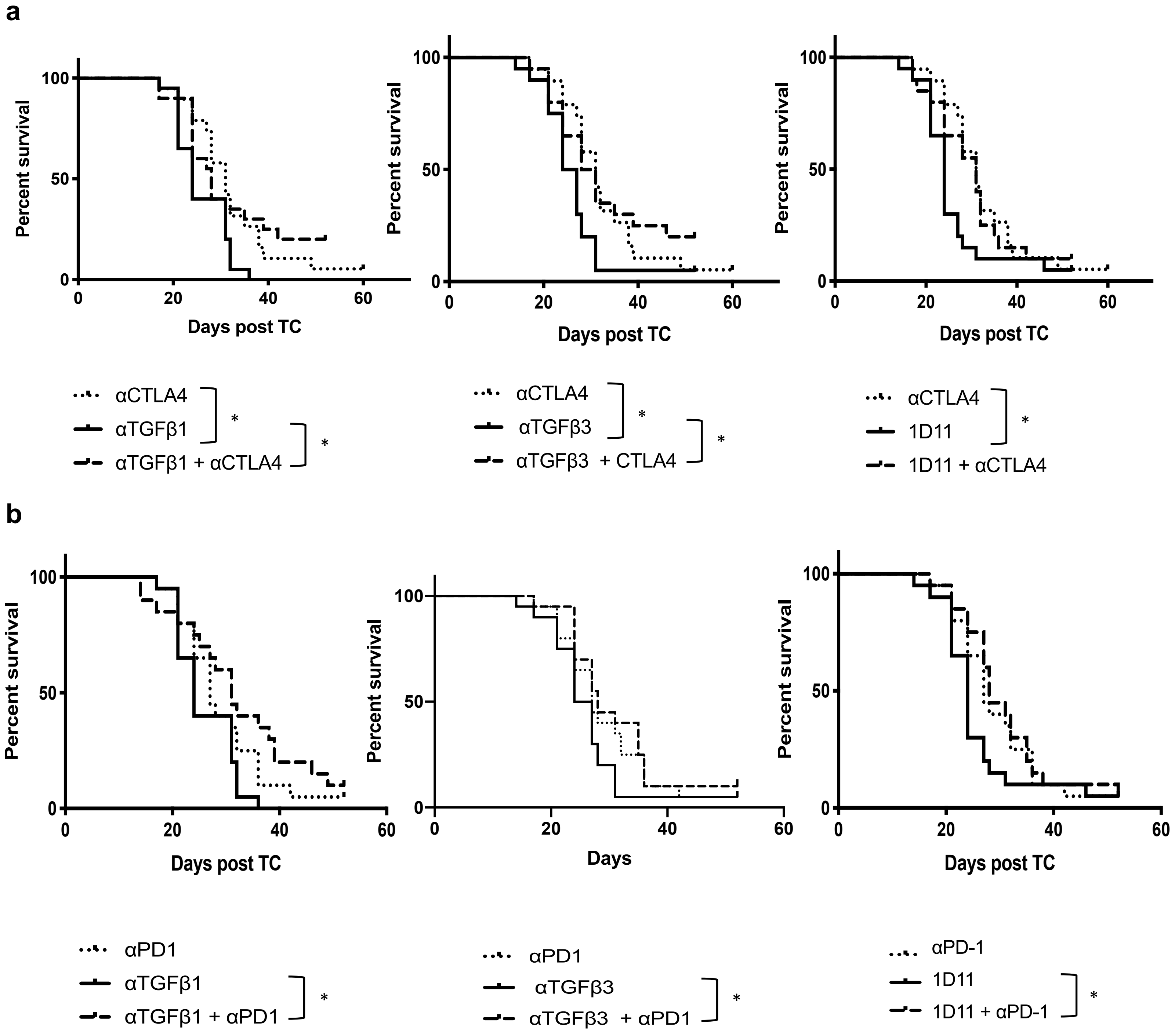

Supplementary figure 12: TGFβ1 and TGFβ3 inhibition in combination with immune checkpoint blockade does not reduce overall survival

Treatment regimen showing the schedule of delivery of anti-TGFβ and anti-CTLA-4 and anti-PD-1 therapy beginning 11 days post tumor implantation is illustrated in Figure 6a. Anti-TGFβ was given via intraperitoneal injection (200 ug/ mouse) every other day for a total of 8 doses. a) Anti-CTLA-4 (clone 9H10) was given via intraperitoneal injection (100 ug/ mouse) every 3 days for a total of 3 doses, Overall survival for indicated treatment combinations with anti-CTLA-4 is shown. b) Anti-PD-1 was given via intraperitoneal injection (250 ug/mouse) every 3 days for a total of 5 doses. Overall survival for indicated treatment combinations with anti-PD-1 is shown. Overall survival curves were derived from pooled values from two independent experiments. p values comparing survival curves were calculated using the Log-rank (Mantel-Cox) test. *$p<0.05$;

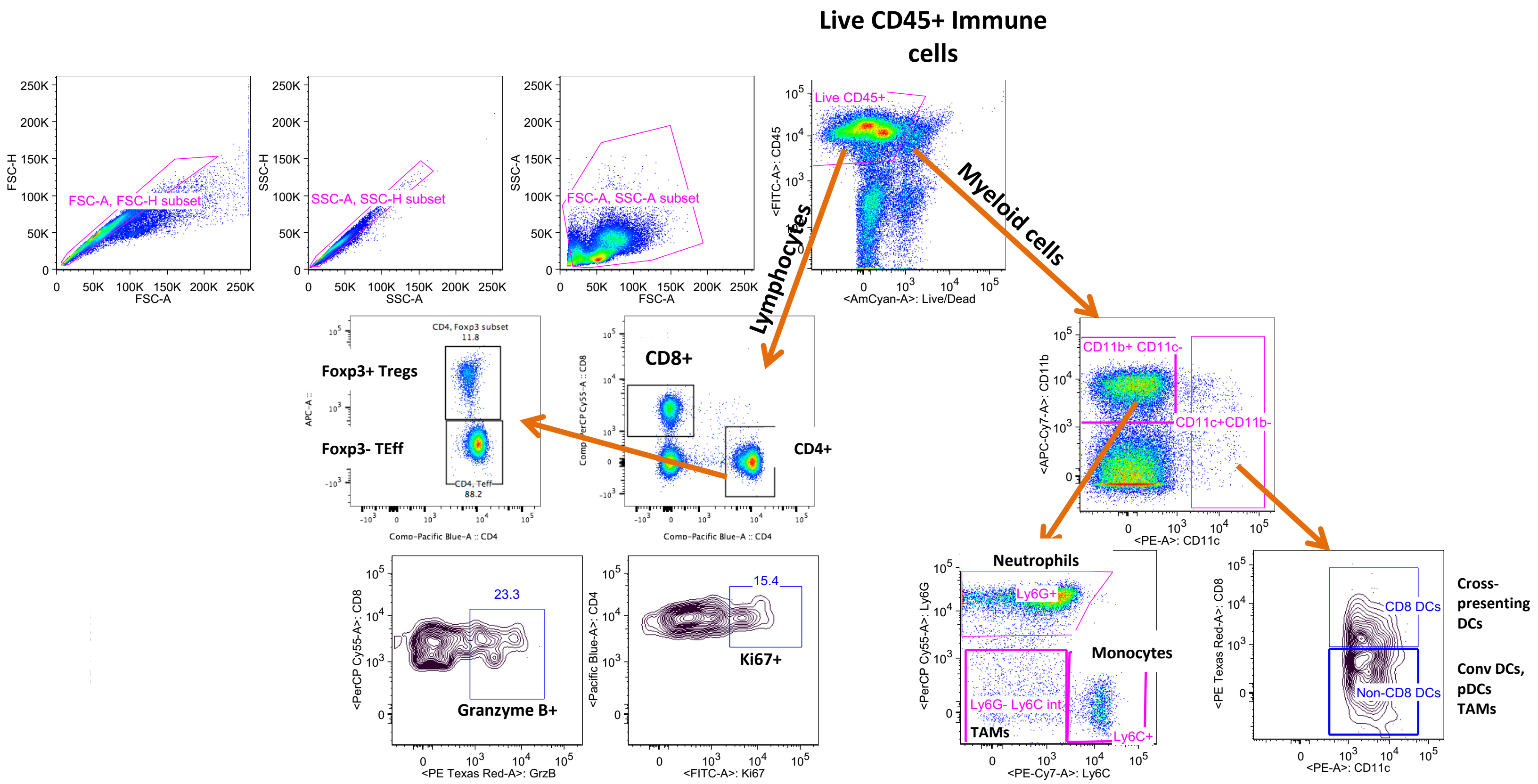

Supplementary figure 13: Gating strategy for flow cytometric analysis

This figure demonstrates the gating strategy used to analyze all flow cytometry data and classify different immune cells. Tregs, Fox3+ CD4+ T regulatory cells; Tefff, Foxp3- C4+ T effector cells; TAMs, tumor associated macrophages; DCs, dendritic cells
