# Supplementary material for: Isoform specific anti-TGFβ therapy enhances antitumor efficacy in mouse models of cancer
Source: Commun Biol. 2021 Nov 17;4:1296. doi: 10.1038/s42003-021-02773-z (PMC8599839; doi:10.1038/s42003-021-02773-z)
Supplement: Supplementary file 3 — Description of Supplementary Files [file 42003_2021_2773_MOESM3_ESM.pdf]

Description of Additional Supplementary Files

File name: Supplementary Data 1

Description: Source data for main Figure files.
