# Supplementary material for: Isoform specific anti-TGFβ therapy enhances antitumor efficacy in mouse models of cancer
Source: Commun Biol. 2021 Nov 17;4:1296. doi: 10.1038/s42003-021-02773-z (PMC8599839; doi:10.1038/s42003-021-02773-z)
Supplement: Supplementary file 5 — Reporting Summary [file 42003_2021_2773_MOESM5_ESM.pdf]

nature research

Corresponding author(s): Jedd Wolchok, Taha Merghoub

Last updated by author(s): Aug 4, 2021

Reporting Summary

Nature Research wishes to improve the reproducibility of the work that we publish. This form provides structure for consistency and transparency in reporting. For further information on Nature Research policies, see our Editorial Policies and the Editorial Policy Checklist.

Statistics

For all statistical analyses, confirm that the following items are present in the figure legend, table legend, main text, or Methods section.

n/a	Confirmed	
☐	☒	The exact sample size (*n*) for each experimental group/condition, given as a discrete number and unit of measurement
☐	☒	A statement on whether measurements were taken from distinct samples or whether the same sample was measured repeatedly
☐	☒	The statistical test(s) used AND whether they are one- or two-sided<br>*Only common tests should be described solely by name; describe more complex techniques in the Methods section.*
☒	☐	A description of all covariates tested
☐	☒	A description of any assumptions or corrections, such as tests of normality and adjustment for multiple comparisons
☐	☒	A full description of the statistical parameters including central tendency (e.g. means) or other basic estimates (e.g. regression coefficient) AND variation (e.g. standard deviation) or associated estimates of uncertainty (e.g. confidence intervals)
☐	☒	For null hypothesis testing, the test statistic (e.g. *F*, *t*, *r*) with confidence intervals, effect sizes, degrees of freedom and *P* value noted<br>*Give P values as exact values whenever suitable.*
☒	☐	For Bayesian analysis, information on the choice of priors and Markov chain Monte Carlo settings
☒	☐	For hierarchical and complex designs, identification of the appropriate level for tests and full reporting of outcomes
☒	☐	Estimates of effect sizes (e.g. Cohen's *d*, Pearson's *r*), indicating how they were calculated

Our web collection on statistics for biologists contains articles on many of the points above.

Software and code

Policy information about availability of computer code

Data collection	No software was used
Data analysis	Flow cytometry data was analyzed using FlowJo software (version 10 - FlowJo, LLC). Image analysis for histologic samples was performed with HALO software Area Quantification module v.1.0 (Indica Labs, Albuquerque, NM) under the supervision of a pathologist. All statistics were calculated using GraphPad Prism software. All software platforms were run on MacOS.

For manuscripts utilizing custom algorithms or software that are central to the research but not yet described in published literature, software must be made available to editors and reviewers. We strongly encourage code deposition in a community repository (e.g. GitHub). See the Nature Research guidelines for submitting code & software for further information.

Data

Policy information about availability of data

All manuscripts must include a data availability statement. This statement should provide the following information, where applicable:
- Accession codes, unique identifiers, or web links for publicly available datasets
- A list of figures that have associated raw data
- A description of any restrictions on data availability

The datasets generated during and/or analysed during the current study are available from the corresponding authors on reasonable request. An Excel file containing all the raw data for the main manuscript figures will be uploaded along with the manuscript. Additional data or analysis will be available upon reasonable request.

Field-specific reporting

Please select the one below that is the best fit for your research. If you are not sure, read the appropriate sections before making your selection.

- [x] Life sciences
- [ ] Behavioural & social sciences
- [ ] Ecological, evolutionary & environmental sciences

For a reference copy of the document with all sections, see nature.com/documents/nr-reporting-summary-flat.pdf

Life sciences study design

All studies must disclose on these points even when the disclosure is negative.

Sample size	Sample sizes of 10 mice per group were utilized to determine treatment efficacy. For the experiments requiring longitudinal measurements of tumor growth, we have consulted with Dr. Katherine Panageas of the MSKCC Biostatistics Service and determined that experimental groups of 10-15 animals are necessary to demonstrate a >50% improvement in tumor-free survival between treatment and control groups without overlapping confidence intervals. The primary endpoint in these experiments is tumor-free progression and time to ≤ 2 cm tumor size. We have shown in our reviewer responses that measurement of tumor size by area and volume yield similar data and relationships. For experiments that require cells from various tissues (spleens, lymph nodes, tumors) for phenotypic/functional analyses we estimate that at least 5 mice/group are needed to allow for assessing differences between groups and generate enough cells to perform T-cell assays.
Data exclusions	Animals with no tumors at the start of treatment or analysis were excluded from experiments.
Replication	All experiments were replicated and the number of times experiments were repeated is indicated in the associated figure legends.
Randomization	Mice were randomized prior to starting treatment to ensure the same average tumor size across treatment groups.
Blinding	The investigators were not blinded to allocation or outcome assessment during experiments.

Reporting for specific materials, systems and methods

We require information from authors about some types of materials, experimental systems and methods used in many studies. Here, indicate whether each material, system or method listed is relevant to your study. If you are not sure if a list item applies to your research, read the appropriate section before selecting a response.

Materials & experimental systems

n/a	Involved in the study	
[ ]	[x]	Antibodies
[ ]	[x]	Eukaryotic cell lines
[x]	[ ]	Palaeontology and archaeology
[ ]	[x]	Animals and other organisms
[x]	[ ]	Human research participants
[x]	[ ]	Clinical data
[x]	[ ]	Dual use research of concern

Methods

n/a	Involved in the study	
[x]	[ ]	ChIP-seq
[ ]	[x]	Flow cytometry
[x]	[ ]	MRI-based neuroimaging

Antibodies

Antibodies used	Anti-TGFβ1 (clone 13A1, mouse IgG1-k) and anti-TGFβ3 (clone 1901 mouse IgG1-k) were provided by the Van Snick laboratory at the Ludwig Institute, Brussels. These antibodies were also custom-ordered from Bioxcell. Anti-PD-1 (clone RMP1-14, catalog # BE0146), anti-CTLA4 (clone 9H10, catalog # BE0131), anti-CD8 (clone 2.43, catalog # BE0061) and anti-TGFβ1,2,3 (clone 1D11.16.8, catalog # BE0057) were purchased from Bioxcell for use in this manuscript.
Validation	An ELISA was used to determine the binding specificities of the anti-TGFβ1 mAb clone 13A1 (IgG1) and anti-TGFβ3 mAb clone 1901 (IgG1) (Uyttenhove, C. et al. 2011) and the specificity of these antibodies is shown in supplementary fig. 1a. These antibodies are not commercially available. For all antibodies purchased from Bioxcell, the manufacturer's website indicates >95% purity as determined by SDS-PAGE for each antibody.

Eukaryotic cell lines

Policy information about cell lines

Cell line source(s)	The B16F10 mouse melanoma line was originally obtained from I. Fidler (MD Anderson Cancer Center, Houston, TX). The 4T1 mouse breast cancer cell line and CT26 colon carcinoma were purchased from ATCC (Manassas, VA). WG492 is a melanoma cell line derived from a tumor from the BRAFV600E/PTEN-/- transgenic mouse.
Authentication	We have authenticated all cell lines used (B16F10, CT26, 4T1 and WG492) in the manuscript via STR profiling and can provide

data upon request.

Mycoplasma contamination	All cell lines routinely tested negative for mycoplasma contamination.
Commonly misidentified lines (See ICLAC register)	No commonly misidentified cell lines were used in this manuscript.

Animals and other organisms

Policy information about studies involving animals; ARRIVE guidelines recommended for reporting animal research

Laboratory animals	C57BL/6J and BALB/cJ mice were purchased from the Jackson Laboratory (Sacramento, CA). All mice used were female between ages 8-10 weeks old.
Wild animals	The study did not involve wild animals.
Field-collected samples	The study did not involve samples collected from the field.
Ethics oversight	Animal experiments were performed in accordance with institutional guidelines under a protocol approved by the Memorial Sloan Kettering Cancer Center (MSKCC) Institutional Animal Care and Use Committee. All mice were maintained in a pathogen-free facility according to the National Institutes of Health Animal Care guidelines.

Note that full information on the approval of the study protocol must also be provided in the manuscript.

Flow Cytometry

Plots

Confirm that:

- [x] The axis labels state the marker and fluorochrome used (e.g. CD4-FITC).
- [x] The axis scales are clearly visible. Include numbers along axes only for bottom left plot of group (a 'group' is an analysis of identical markers).
- [x] All plots are contour plots with outliers or pseudocolor plots.
- [x] A numerical value for number of cells or percentage (with statistics) is provided.

Methodology

Sample preparation	C57BL/6 female mice were tumor challenged and treated as described in the methods section of the manuscript. At different time points post tumor challenge, mice were euthanized and B16F10 tumors and corresponding spleens (if required) were harvested. Single cell suspensions were prepared by mechanical dissociation through 40 µM cell strainers and red blood cells were removed from spleens using ACK lysis buffer (Lonza, Walkersville, MD). For staining for flow cytometry analysis: 100 µL of single cell suspensions of each tissue were plated in 96-well round bottom plates. Cells were pelleted by spinning at 2,000 RPM for 5 mins then incubated in 100 µL of 5µg/mL Fc-block antibody (clone 2.4G2) for 20 mins on ice in FACS buffer (PBS + 0.5% BSA + 2mM EDTA). After Fc-block, cells were stained in FACS buffer containing fluorophore conjugated surface antibodies and a fixable viability dye (efluor506, eBioscience) for 20 mins on ice, then washed two times with 200 µL FACS buffer. All intracellular staining was conducted using the Foxp3 fixation/permeabilization buffer according to the manufacturer’s instructions (eBioscience).
Instrument	The samples were acquired on a LSRII (BD Biosciences) or Cytek Aurora using optimized fluorophore panels.
Software	Data was analyzed using FlowJo software (version 10 - FlowJo, LLC) run on MacOS. Data was plotted and statistics were calculated using GraphPad Prism software to conduct ANOVA and two-tailed Student's T test.
Cell population abundance	Cell population abundance was determined via the gating strategy outlined below. Cells were not sorted.
Gating strategy	Cells were first gated based on FSC and SSC gates. Cells were then gated based on live and CD45+. They were then gated based on lymphoid populations of CD8+ and CD4+, which was further divided in FoxP3+ T regulatory cells and FoxP3- T effector cells. CD8+ cells were then gated on PD1+, Ki67+ and Granzyme B+ with positive cells based on florescence minus one (FMO). Myeloid cells were gated on CD11b+ and CD11c+ with an overlapping population noted that is both CD11b+ and CD11c+. CD11b+ cells were further gated based on Ly6G+ (neutrophils), Ly6C high (monocytes), which was further subdivided into F4/80+ (macrophages). CD11c+ cells were gated based on CD8+ dendritic cells or CD11b+ or CD11b- dendritic cells. Cells expressing TGFbeta were gated based on an FMO. A diagram demonstrating our gating strategy is included as a supplementary figure.

- [x] Tick this box to confirm that a figure exemplifying the gating strategy is provided in the Supplementary Information.
